# Sorting and identification of side population cells in the human cervical cancer cell line HeLa

**DOI:** 10.1186/1475-2867-14-3

**Published:** 2014-01-13

**Authors:** Wenjuan Qi, Chao Zhao, Lijun Zhao, Ning Liu, Xiaoping Li, Weidong Yu, Lihui Wei

**Affiliations:** 1 Department of Gynecology and Obstetrics, Peking University People's Hospital, 11 Xizhimen South Street, Xicheng District, Beijing 100044, China; 2 Central Laboratory, Peking University People's Hospital, 11 Xizhimen South Street, Xicheng District, Beijing 100044, China; 3 Haidian Maternal and Child Health Hospital, 33 Haidian South Road, Haidian District, Beijing 100080, China

**Keywords:** Cervical cancer cells, Side population cells, Fluorescence-activated cell sorting, Chemoresistance, Radioresistance

## Abstract

**Background:**

Several reports have revealed that cancer stem cells (CSCs) exist in many types of solid tumors. Some studies have demonstrated that side population (SP) cells isolated from diverse cancer lines harbor cancer stem-like properties, but there are few reports examining the characteristic of SP cells in human cervical cancer. The aim of this study is 1) to find out a feasible way to detect the tumor stem-like cells in cervical cancer, and 2) to analyze the properties of the SP cells being sorted.

**Methods:**

Isolated SP and non-SP cells from human cervical cancer cell line Hela by Hoechst 33342 dying method and flow cytometry analysis. Observing morphology of SP and non-SP cells. The expression of various biomarkers putatively related to cancer stem cells were investigated by immucytochemistry of SP and non-SP cells. We also analyzed cell cycle and cell apoptosis for sorted cells. The oncogenicity of the SP and non-SP cells were analyzed by tumor formation in nonobesediabeti- c/severe combined immune- deficient (NOD/SCID) mice. The drug-resistant and radiation-resistant index between SP, non-SP and Hela cells was estimated by MTS assay.

**Results:**

The fraction of SP cells in Hela was approximately 1.07 ± 0.32%. SP cells were smaller and rounder in shape than non-SP cells, and mostly showed colony-like growth. Immunocytochemistry showed that stem cell makers (Oct3/4, CD133, BCRP) were highly expressed in SP cells. Moreover, the number of apoptotic cells among non-SP cells (17.6 ± 3.7%) was significantly higher compared with that among SP cells (4.4 ± 1.2%). The HE staining of in vivo grown tumors result from SP cells showed more poor differentiation, though no significant differences were shown between SP and non-SP cells in NOD/SCID mice tumorigenicity. Furthermore, SP cells demonstrated a higher degree of drug resistance against trichostatin A (TSA) compared with that of non-SP and Hela cells. SP cells were also found to be more resistant against radiotherapy.

**Conclusions:**

SP cells possess some characteristics of CSCs, namely high proliferation ability, chemoresistance and radioresistance, which may be helpful to elucidate novel targets for effective clinical treatments of cervical cancer in the future.

## Background

Cervical cancer is a common gynecological malignancy. Persistent human papilloma virus infection has been recognized as the primary pathogenic factor for the development of cervical cancer, however its mechanism remains unclear. Recent studies have shown that tumor tissues contain a very small number of stem-like cells that are responsible for self-renewal, differentiation, tumor growth, metastasis and recurrence [1,2]. Hence, an increasing number of studies have been conducted on cancer stem cells (CSCs) in an attempt to identify the mechanisms of the genesis, development and drug resistance of tumors. The principal problem of the relevant research is the isolation and identification of CSCs. Because of lack of markers, CSCs have been mainly isolated by approach of isolating adult stem cells. These methods include cell sorting based on the expression of surface biomarkers, suspension sphere culture, and functional cell sorting based on the biological characteristics of the cells (side population (SP) cell sorting).

In 1996, Goodell et al. [3] discovered SP cells when examining mouse bone marrow hematopoietic stem cells using the fluorescent dye Hoechst 33342. After more than a decade of research, SP cells are considered to be a common phenotype of stem cells. To date, SP cells have been isolated from many tumor tissues and cell lines such as blood [4], breast [5], glioma [5], cervix [5], liver [6], ovarian [7], lung [8], and pancreas [9]. Further experiments have confirmed that SP cells possess CSC-like features including self-renewal, asymmetric division into SP and non-SP cells, and apparent drug resistance. Many reports indicate that SP cells are an ideal model for stem cell research [10]. The characteristic of SP cells to rapidly extrude Hoechst 33342 is based on the expression of ABCG2/BCRP1, a breast cancer resistance protein (BCRP) of the ATP-binding cassette (ABC) transporter family. ABCG2/BCRP1 is a transmembrane protein that plays an important role in the multidrug resistance (MDR) of tumor cells [11]. The expression of ABCG2/BCRP1 shows a strong positive correlation with the phenotype of SP cells in a series of studies, which is the molecular basis of the phenotypic characteristics of SP cells [12]. High expression of ABCG2/BCRP1 in SP cells is contributed to drug resistance and tumor recurrence [10].

In 2004, Kondo et al. [5] reported that the SP cells isolated from the cervical cancer cell line HeLa account for approximately 1.2% of the total number of HeLa cells. However, few studies have reported on the phenotypic identification of SP cells among HeLa cells. The present study attempts to find an effective method for the isolation of cervical CSCs. We sorted and cultured SP cells from the cervical cancer cell line HeLa, and then identified their stem-like characteristics. This study may be helpful to elucidate novel targets for effective clinical treatments of cervical cancer.

## Results

### SP cells among HeLa cells

**Figure 1:**
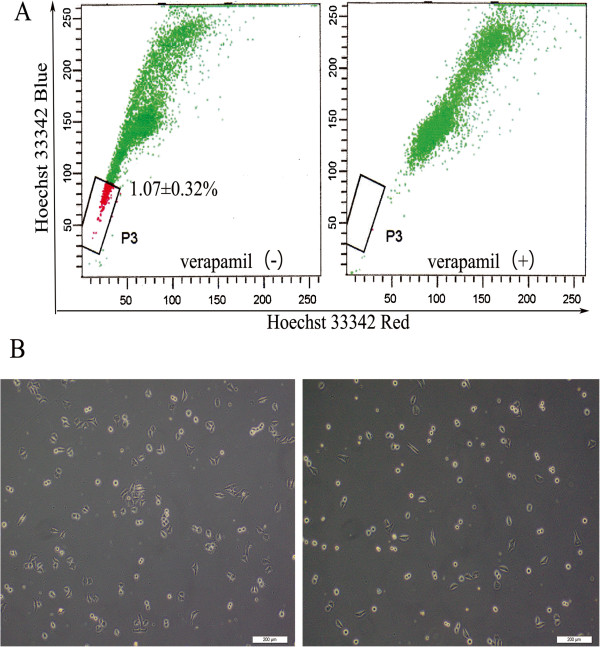
**Cell sorting results and morphological observation. (A)** Sorting of SP cells using Hoechst 33342 (1.07 ± 0.32% to total cells) (left). When the cells preincubated with verapamil to block the ATP transporter, the SP proportion was reduced to 0 (right). **(B)** After plating, SP and non-SP cells were observed every 6 hour, SP cells were more adherent than non-SP cells and showed colony-like growth. The image was obtained in 24 hour (×100).

After excluding dead cells and cellular debris based on scatter signals and propidium iodide (PI) fluorescence, the SP and non-SP cells were sorted. In multiple independent HeLa cultures, we detected 1.07 ± 0.32% SP cells as shown in Figure 1A.

After 6 hour plating, observation of the morphology of SP and non-SP cells revealed that the SP cells were more adherent than non-SP cells. In addition, the sorted SP cells were smaller and rounder in shape than non-SP cells, and mostly showed colony-like growth (Figure 1B).

### Expression of various biomarkers related to stem cells

**Figure 2:**
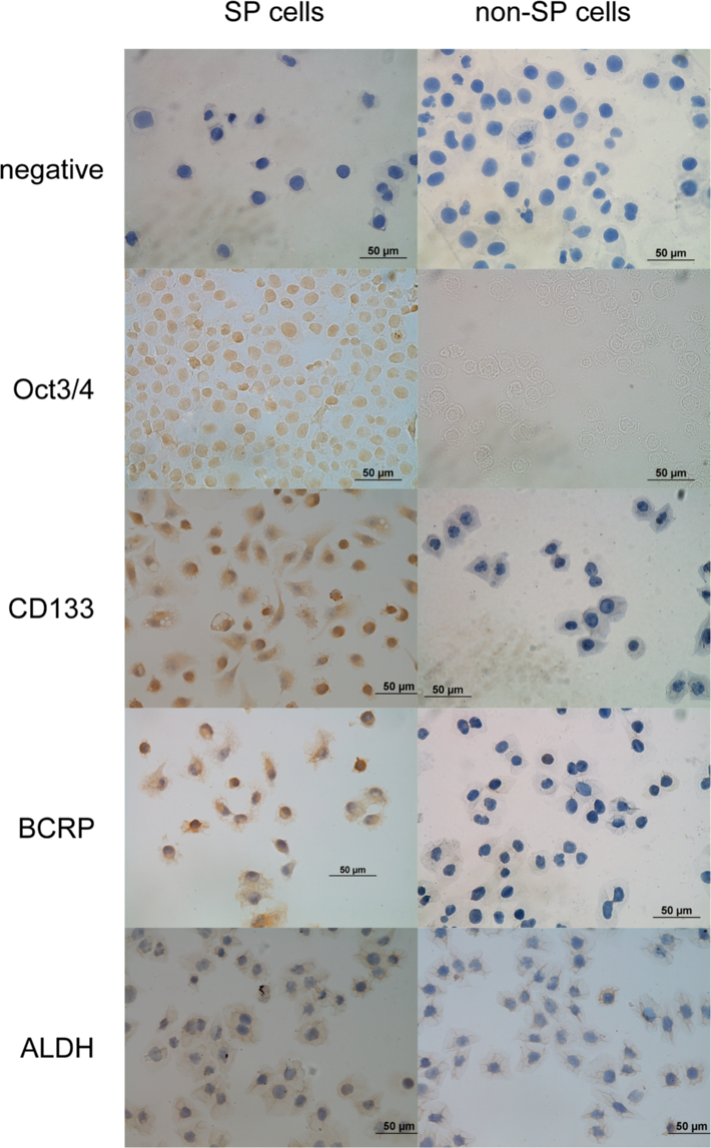
**Expression of putatively CSCs related markers (×400 magnification).** Compared to negative control, Oct3/4, CD133 and BCRP expression in SP cells were higher than non-SP cells. BCRP was scarcely expressed in non-SP cells. ALDH-1 expression showed no visible difference between SP and non-SP cells (×400).

The expression of various biomarkers putatively related to CSCs was investigated in freshly sorted SP and non-SP fractions of HeLa cells (Figure 2). Considering that the expression of Oct3/4 mainly occurs in the nucleus, hematoxylin counterstaining of the nucleus was omitted in the immunocytochemical analysis. The results showed that Oct3/4 was mainly expressed in the nucleus, and a small amount of Oct3/4 was found in the cytoplasm. The expression level of Oct3/4 in SP cells was substantially higher than that in non-SP cells. CD133 and BCRP were mainly expressed in the cytoplasm, and their expression levels were substantially higher in SP cells compared with non-SP cells. Notably, BCRP was hardly expressed in non-SP cells. ALDH-1 was expressed in the cytoplasm of SP and non-SP cells, and the expression level was almost the same in SP and non-SP cells. These results indicate that the phenotype of SP cells is closely related to the expression of BCRP and shows a certain correlation with stem cell-related biomarkers, i.e., Oct3/4 and CD133.

### Cell cycle and apoptosis analyses

**Figure 3:**
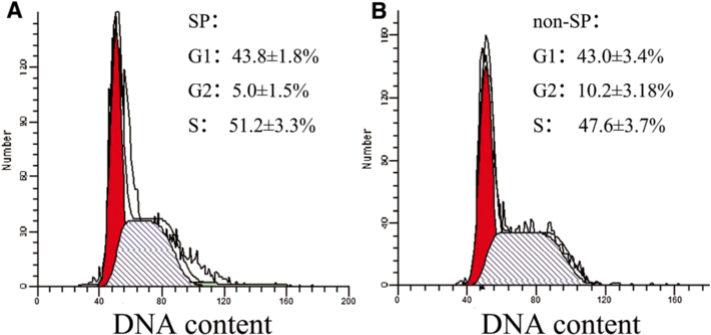
**Cell cycle of SP and non-SP cells.** Cell cycle analysis of sorted SP **(A)** and non-SP **(B)** at 24 hours after fluorescence-activated cell sorting isolation. The results revealed no significant difference between SP and non-SP cells.

We analyzed the cell cycle of SP and non-SP cells sorted from HeLa cell line. No significant difference was observed in the cell cycle distribution between SP and non-SP cells under normal culture conditions. (G1: 43.8 ± 1.8% vs. 43.0 ± 3.4%, *P* = 0.78; G2: 5.0 ± 1.5% vs. 10.2 ± 3.18%, *P* = 0.12; S: 51.2 ± 3.3% vs. 46.8 ± 5.6%, *P* = 0.40; n = 3) (Figure 3).

**Figure 4:**
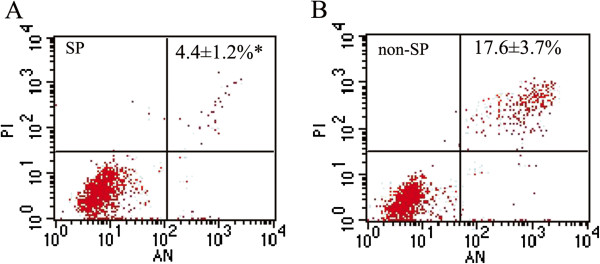
**Cell apoptosis analysis of SP and non-SP cells.** Cell apoptosis analysis showed that the apoptotic rate of SP cells **(A)** was apparently lower than that of non-SP cells **(B)**.

We also detected apoptosis by annexin V-PI staining and flow cytometry at 24 hour after FACS isolation. As shown in Figure 4, Table 1 the apoptotic rate of non-SP cells (17.6 ± 3.7%) was significantly higher than that of SP cells (4.4 ± 1.2%, *P* = 0.004; n = 3), and the active cells in SP cells were apparently more than non-SP cells, which indicated that the anti-apoptosis ability of SP cells was more efficient (Table 1, Figure 4).

**Table 1:** Apoptosis analysis of SP and non-SP cells

	**SP cell (%)**	**Non-SP cell (%)**
Active cell	95.1 ± 1.4%	84.2 ± 3.6%*
Early apoptosis	1.2 ± 0.6%	2.9 ± 1.6%
Late apoptosis	3.2 ± 0.8%	14.7 ± 5.2%
Necrotic cell	0.5 ± 0.3%	1.5 ± 1.6%
Early–late apoptosis	4.4 ± 1.2%	17.6 ± 3.7%*

*P < 0.05 t-test.

### Tumor formation in NOD/SCID mice

**Figure 5:**
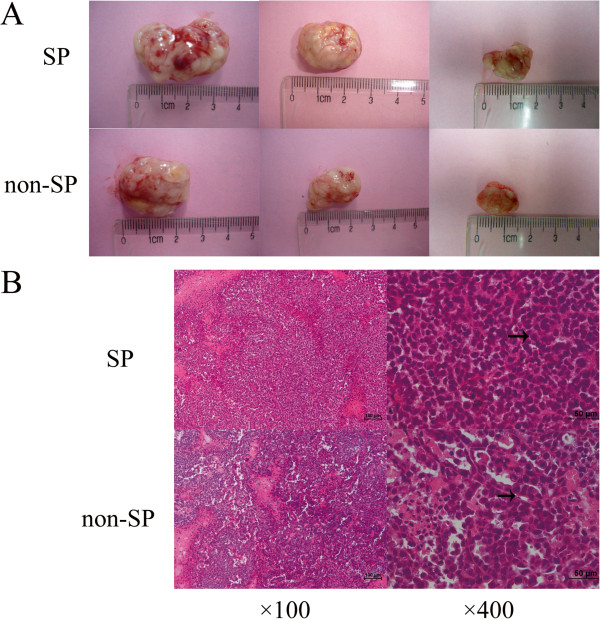
**Tumor formation in NOD/SCID mice and H&E staining result. (A)** After inoculated with 1 × 10^5^ (left), 1 × 10^4^ (middle)and 2 × 10^3^ (right) SP or non-SP cells to NOD/SCID mice, it seemed no statistically significant differences in tumorigenicity, i.e. incidence, latency and growth rate between SP and non-SP cells. **(B)** Representative H&E stained photomicrographs of SP and non-SP tumors. The tumor resulting from non-SP cell was similar to cervical adenocarcinoma, and it contained duct lumen. As for tumor induced by SP cells, tissue lost their typical characteristics of adenocarcinoma with more obvious cell atypia and fewer duct lumen.

We tested the tumorigenic potential of SP and non-SP cells by tumor incidence, latency (i.e., the time between tumor cell implantation and when tumors can first be palpated), and growth rate (i.e., tumor volume). It was evident that with an decreasing number of injected cells, the tumor incidence in NOD/SCID mice decreased, while the latency of tumorigenesis was noticably prolonged and the tumor volume gradually decreased. However, there were no statistically significant differences in the above-mentioned parameters between SP and non-SP cells (Table 2, Figure 5A). The t-test showed no statistical differences in tumor latency and volume between mice inoculated with 1 × 10^5^ SP and non-SP cells. Fisher’s exact test showed no statistical differences in tumor incidence between mice inoculated with 1 × 10^4^ or 2 × 10^3^ cells. No statistical analysis was performed on data of the tumor latency and volume in mice inoculated with 1 × 10^4^ or 2 × 10^3^ cells because of the insufficient number of samples (n < 3). Hematoxylin and eosin (H&E) staining was performed to demonstrate that the xenografts in immunodeficient mice were generated from the injected human HeLa cells. We found that the tumor result from SP cell injection was poorer differentiation (Figure 5B).

**Table 2:** Tumorigenic potential of SP and non-SP cells in NOD/SCID mice

	**Incidence**		**Latency (day)**		**Volume (cm**^**3**^**)**	
**Cell no**	**Non-SP**	**SP**	**Non-SP**	**SP**	**Non-SP**	**SP**
1 × 10^5^	3/3	3/3	20.67 ± 2.89	20.00 ± 3.61	5.22 ± 2.12	4.38 ± 1.14
1 × 10^4^	2/3	2/3	30.00 ± 4.24	23.00 ± 4.24	3.32 ± 3.50	3.63 ± 3.60
1 × 10^3^	2/3	1/3	33.5 ± 9.19	37	1.18 ± 0.72	1.37

### SP cells exhibit increased resistance against TSA (Trichostatin A)

**Figure 6:**
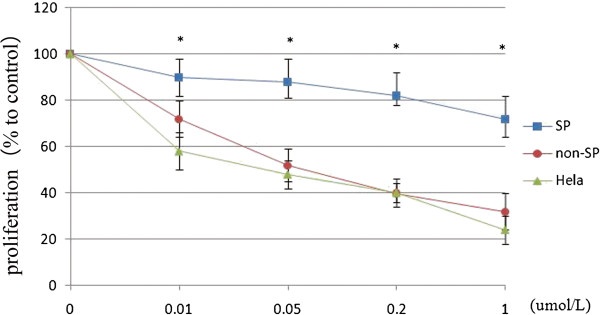
**Chemotherapy sensitivity assays of SP and non-SP cells.** Growth inhibition effect of TSA on sorted SP, non-SP cells, and unsorted HeLa cells. After 72 h of TSA treatment at various concentrations, unsorted HeLa cells and non-SP cells showed substantially suppressed growth in a dose-dependent manner, whereas SP cells were unaffected. Data are presented as the means of three separate experiments, each performed in triplicate. *P < 0.01, t-test.

Hela, SP and non-SP cells were treated with varying concentrations of TSA. Even at 0.01 μmol/L TSA, the viability of SP cells was clearly higher than that of non-SP cells. As doses of TSA increased, the growth of HeLa and non-SP cells was obviously suppressed. The suppressive effect reached the peak when cells were treated with 0.2 μmol/LTSA. The SF (surviving fraction) of sorted SP cells (86.68 ± 8.78%) was significantly higher than that of non-SP (49.06 ± 6.26%) and unsorted HeLa cells (43.69 ± 4.84%) (*P* < 0.05). However, TSA had no significant suppressive effect on the growth of SP cells (Figure 6). These results demonstrate the apparent chemoresistance of HeLa stem-like cells against anticancer drugs, which may contribute to tumor recurrence and MDR.

The SF (surviving fraction) of HeLa, SP and non-SP cells was calculated as follows: SF = experiment OD/control OD.

### Radiation sensitivity

**Figure 7:**
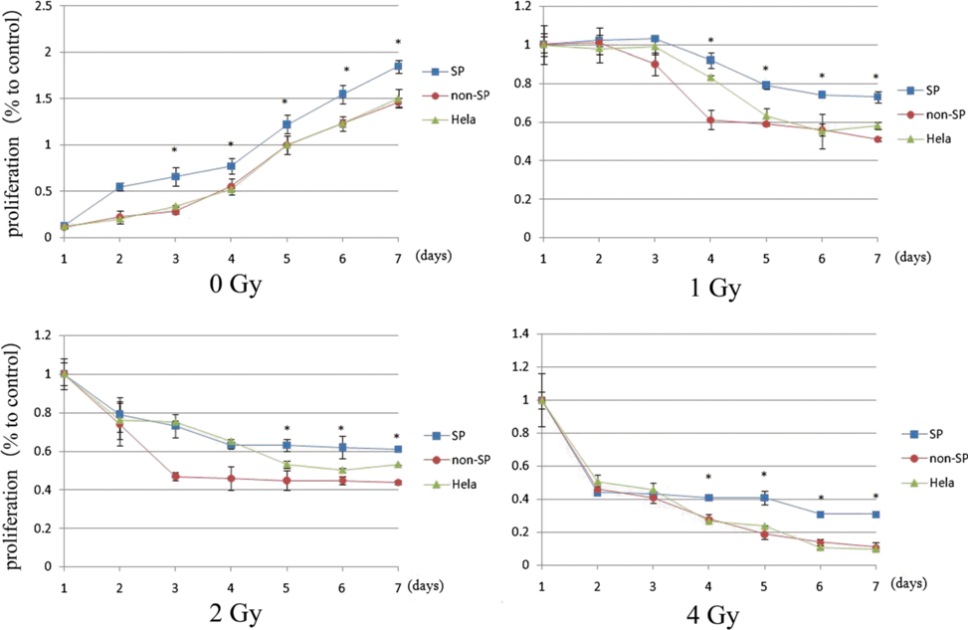
**Radiation sensitivity assays of SP, non-SP and Hela cells.** SP, non-SP and Hela Cells irradiated at various doses. After 7-day incubation, significant difference were detected between SP and non-SP cells and between SP and Hela cells. Data are presented as the means of three separate experiments, each performed in triplicate. *P < 0.01, t-test.

To examine whether the SP cells from the HeLa cell line possess a radioresistant phenotype, we exposed SP, non-SP and HeLa cells to X-rays to determine their sensitivity to radiation. After irradiation, we cultured the cells for 7 days, and then subjected them to an MTS assay. All the cell types showed sensitivities to X-ray irradiation, and their cell proliferation rates decreased with increasing doses of radiation. Exposure to X-rays at 1, 2, or 4 Gy, the SFs of SP, non-SP and HeLa cells were resulted in significant differences. As shown in Figure 7, SP cells grew faster than non-SP cells when they were exposed to different does X ray. SP cells showed great radioresistance than the other cells. On the 7th day after irradiation, the SFs of SP, non-SP and HeLa cells were as follows respectively: 1 Gy, 0.73 ± 0.25 vs. 0.51 ± 0.14 vs. 0.58 ± 0.15; 2 Gy, 0.61 ± 0.11 vs. 0.44 ± 0.12 vs. 0.53 ± 0; and 4 Gy, 0.31 ± 0.02 vs. 0.11 ± 0.02 vs. 0.1 ± 0.

## Discussion

Since the stem cell theory of cancer was proposed, it was first confirmed in the field of hematology [13]. Isolation of CSCs from solid tumors is usually performed by cell sorting based on the expression of putative surface biomarkers of stem cells. Recent studies of stem cells have shown that a small population of cells can specifically extrude the DNA dye Hoechst 33342. Such cells show weak fluorescence in flow cytometry, and have been named as SP cells. Further studies reported that SP cells are found in several cancer cell lines, and demonstrate certain stem cell-like phenotypic characteristics [14-17]. Hence, it is very likely that SP cells include highly tumorigenic stem-like cells, which provides a practical method for preliminary identification and sorting of CSCs. Compared with cell sorting through surface biomarkers, sorting of SP cells is more convenient, less costly, and universally applicable. Moreover, the latter can be used to isolate SP cells with unknown surface biomarkers [18]. Taken together, in the present study, we used FACS to isolate SP cells from the human cervical cancer cell line HeLa, and then examined the biological characteristics of the sorted SP cells.

In 2004, Kondo et al. [5] isolated SP cells from the HeLa cell line, which accounted for approximately 1.2% of the total number of HeLa cells. In our study, we obtained sorted SP cells that accounted for 1.07% of the total number of HeLa cells. The result of SP cells in this study was mostly consistent with Kondo's. Microscopic observations showed that the sorted SP cells shared morphological characteristics with stem-like cells, including smaller size, rounder shape and higher adherence than non-SP cells, as well as colony-like growth. This result suggests that SP cells may possess certain stem cell morphological characteristics.

In addition, we tested the expression of surface markers of embryonic stem cells and hematopoietic stem cells in SP and non-SP cells. The expression levels of Oct3/4 and CD133 in SP cells were significantly higher than those in non-SP cells, which is consistent with previous findings [19,20]. As suggested in many previous studies, high expression of ABCG2/BCRP on the cell membrane is a prerequisite for SP cells to extrude Hoechst 33342 dye and maintain their stem cell-like characteristics [21-23]. In our study, immunocytochemical analysis showed that BCRP was highly expressed in the sorted SP cells, whereas non-SP cells hardly expressed BCRP, which is consistent with the proposed molecular mechanism mentioned above.

In addition to those surface markers, ALDH-1 (aldehyde dehydrogenase 1, ALDH1, ALDH1A1 or RALDH1) has received increasing attention as a specific marker for CSCs. ALDH-1 of the ALDH family is a cytosolic enzyme that catalyzes the intracellular oxidation of acetaldehyde to acetate, and is involved in the differentiation and gene expression of a variety of tissues. It is also a specific marker of normal stem cells in tissues. In recent years, breast, lung, prostate, and pancreatic cancers have been found to be associated with high expression of ALDH-1 in a small number of cells with stem cell-like characteristics [24-27]. In our study, no significant differences were observed in the expression of ALDH1 between sorted SP and non-SP cells (Figure 2), which might due to the different origin and differentiation of tumor cells. Further investigation is needed to treat ALDH-1 as a cell surface stemness-related marker of cervical cancer cells.

According to the stem cell theory of cancer, CSCs have the potential for continuous differentiation and self-renewal. SP cells can give rise to both SP and non-SP cells through asymmetric division, whereas non-SP cells can only differentiate into non-SP cells [28,29], resulting in higher tumorigenicity of SP cells than non-SP cells. In our experiment, however, no significant differences were observed in the tumorigenicity between SP and non-SP cells at doses of 1 × 10^5^, 1 × 10^4^, and 2 × 10^3^ cells per mouse. This result is inconsistent with asymmetric division of SP cells and the previous findings in several reports [29-31]. Several recent studies have also challenged the conventional theory of asymmetric division. In non-small cell lung cancer, Pan et al. [32] found that SP and non-SP cells can both further divide into SP and non-SP cells. In another study, SP cells were obtained by re-sorting non-SP cells isolated from C6 glioma cells and then culturing them in a serum-containing medium for 2 weeks [33]. Furthermore, a model has been proposed in which transformation between SP and non-SP cells can be achieved through a shift in the localization of ABCG2/BCRP between the cell membrane and cytoplasm [22]. Therefore, in our study, transplanted non-SP cells may have produced SP cells, leading to no significant difference in the tumorigenicity of the two subpopulations of cells. But the HE staining result, however, confirmed that the differentiation ability was different between SP and non-SP cells.

CSCs have the potential for self-renewal and continuous differentiation. More importantly, these cells are more resistant against radiotherapy and chemotherapy, which is the most plausible reason for the failure of clinical treatments of cancer.

In the present study, comparison of the radioresistance and chemoresistance among HeLa, SP and non-SP cells showed that SP cells were more resistant against radiotherapy and chemotherapy than HeLa and non-SP cells. As described above, high expression of ABCG2/BCRP on the SP cell membrane is the molecular basis for FACS of SP cells. ABCG2/BCRP pumps out not only the Hoechst 33342 dye, but also relevant metabolites, drugs, and toxic substances [34], thereby constituting the molecular mechanism for the drug resistance of SP cells.

One recent study from Xia P et al. [35] found some characteristics of SP and non-SP cells have changed after ionizing radiation. Protein levels of Bcl-2 and Bcl-xl were decreased, while Bax expression was increased in non-SP cells following radiation exposure. In addition, increased activation of caspase-3 and caspase-9 were detected after radiation exposure in non-SP cells.

In our research, the apoptosis was 2 ~ 4 times higher in non-SP than SP cells without radiation. This is not the direct cause of less proliferation after chemotherapy treatment and radiation in non-SP cells, however. In the research of Xia P et al. [35] the apoptosis rate of non-SP cells exposed to 8 Gy radiation was 22.9% ± 0.43%, whereas no change in the SP cells at the same dose exposure. Some pathway may be involved in the decreasing proliferation, increasing apoptosis and mitochondria damage after chemotherapy and radiation in non-SP, but not in SP cells.

In further studies of ABCG2/BCRP, an increasing number of researchers have attempted to identify the pathways involved in BCRP-mediated radioresistance and chemoresistance. At present, it is widely believed that the expression of ABCG2/BCRP on the cell surface is positively correlated with activation of the PI3K/Akt pathway. Liang [36] and Zhang [37] have shown that the PI3K/Akt signaling pathway affects tumor radioresistance by anti-apoptosis and activation of DNA repair mechanisms. Moreover, in a study of malignant glioma, Keishi [38] found that the PI3K/Akt signaling pathway affects the radioresistance of tumor cells by mediating the autophagy process.

So far, it still remains far from clear for the exact mechanisms of the radioresistance and chemoresistance of SP cells and further investigations are needed.

## Conclusion

In summary, a small number of SP cells were sorted from the HeLa cervical cancer cell line, which showed strong capacities for proliferation, anti-apoptosis, and certain degrees of radioresistance and chemoresistance. These SP cells likely include stem-like tumor cells. Further study of the sorted SP cells from HeLa cells may provide new insights into the treatment of cervical cancer.

## Materials and methods

### Cell culture

The human cervical cancer cell line HeLa was purchased from the American Type Culture Collection and maintained in the laboratory of the Department of Gynecology and Obstetrics, Peking University People's Hospital (Beijing, China). HeLa cells were maintained as adherent monolayer cultures in high-glucose Dulbecco Modified Eagle's Medium (DMEM, HyClone, USA) supplemented with 10% fetal bovine serum (FBS, HyClone, USA) and incubated at 37°C with 5% CO_2_. The medium was replaced every 2–3 days. At 80–90% confluency, the cells were washed twice with PBS, and then digested with 0.25% trypsin (Sigma Aldrich, USA) and 0.02% EDTA (Amresco, USA) (v/v, 1:3).

### Fluorescence-activated cell sorting of SP cells

HeLa cells in the logarithmic growth phase were trypsinized, washed twice with PBS, and counted. Then, the HeLa cells were resuspended in DMEM with 2% FBS (5 × 10^6^ cells/mL) and divided into two groups. Group 1 was incubated with the DNA binding dye Hoechst 33342 (Sigma Aldrich, USA) at a final concentration of 5 ug/mL for 90 min at 37°C with gentle agitation every 15 min. Group 2 was pretreated with 50 μg/mL verapamil (Sigma Aldrich, USA) for 15 min at 37°C, and then incubated with Hoechst 33342 (final concentration: 5 μg/mL) for 90 min at 37°C with gentle agitation every 15 min. The incubation process was carried out in the dark. The cells were then washed twice with ice-cold PBS and resuspended in PBS containing 2% FBS and 10 mM HEPES. The cell suspension was stored at 4°C while protected from light before FACS. Cell suspensions were freshly prepared for cell sorting and stained with PI (Sigma Aldrich, USA) at a final concentration of 2 μg/mL. Cell sorting was performed using a FACS DIVA fluorescence-activated cell sorter (BD Biosciences, USA). SP and non-SP cells were collected separately in sterile 25-cm^2^ flasks and cultured in DMEM containing 10% FBS at 37°C with 5% CO_2_. Cell morphology was examined under an inverted microscope every 6 h.

### Immunocytochemistry

To determine whether there were expression differences of stem-like cell biomarkers between SP and non-SP cells, freshly sorted SP and non-SP cells were cultured in chamber slides under normal culture conditions overnight. The cells were then fixed with 4% paraformaldehyde for 30 min at room temperature, treated with 2% H_2_O_2_ for 30 min followed by 0.3% Triton X-100 for 30 min, and then stained with the following antibodies: anti-Oct3/4 (mouse monoclonal, 1:400; ZSGB-BIO, China), anti-CD133 (rabbit polyclonal, 1:1200; BioSS, China), anti-BCRP (mouse monoclonal, 1:800; Abcam, UK), and anti-ALDH (rabbit polyclonal, 1:1100; BioSS, China). The negative control omitted the primary antibody. Cells were incubated in a humidified box at 4°C overnight, and then the secondary antibody was added to the cells, followed by incubation at room temperature for 30 min. After the reaction with DAB, the cells were smeared onto slides, examined by microscopy and photographed with a digital camera connected to the microscope.

### Cell cycle and apoptosis analyses

Cell cycle and apoptosis analyses were both performed after 24 hours of sorting.

The cell cycle was examined using a CycleTEST™ PLUS DNA Reagent Kit (BD Biosciences, USA) following the manufacturer’s instructions.

Cell apoptosis was examined using an Annexin V-FITC Apoptosis Detection Kit (BSCs) following the manufacturer’s instructions. Briefly, the cells were counted and 5 × 10^5^~1 × 10^6^ cells of each group were centrifuged at 179 *g* (4°C) for 10 min. After the supernatant was removed, cold PBS was added to the cell pellet, followed by gentle vortexing to resuspend the cells. Then, the cells were washed twice, resuspended in 200 μL binding buffer containing 10 μL annexin V-FITC, and gently mixed and incubated at room temperature for 15 min while protected from light. Finally, 300 μL binding buffer and 50 μL PI were added to the cell suspension, followed by flow cytometric analysis. Each sample was prepared in triplicate.

### *In vivo* xenografting in immunodeficient mice

NOD/SCID mice were purchased from the Animal Institute of the Chinese Academy of Medical Science (CAMS) and Peking Union Medical College (PUMC) (Certificate No. SCXK(jing)2009-0004), and maintained in microisolator cages. All experiments were approved by the Animal Care Committee of CAMS and PUMC. Freshly sorted SP and non-SP cells in 200 μl Matrigel (BD Biosciences, USA) diluted in PBS at a 1:1 ratio were injected subcutaneously into the left axillary fossa of female NOD/SCID mice (4–6 weeks-old). Groups of mice were inoculated with SP or non-SP cells at 1 × 10^5^, 1 × 10^4^ and 2 × 10^3^ cells, respectively. Tumor appearance was inspected weekly by visual observation and palpation. Mice were sacrificed after 8 weeks and the tumors were harvested, measured, and photographed. Tumor volumes were measured using a digital caliper and approximated according to the formula V = 1 / 2ab^2^, where a and b are the long and short diameters of the tumor, respectively [30]. Tumors were fixed in 10% buffered formalin, embedded in paraffin, and then sections were prepared for H&E staining.

### Chemoresistance analysis

The sensitivities to chemotherapeutic reagents of HeLa, SP, and non-SP cells were assessed using an MTS assay. Briefly, 2 × 10^3^ cells per well were seeded on 96-well plates in 200 μl per well of appropriate growth medium. After 24 hours, the cells were treated with TSA at various concentrations (0.01, 0.05, 0.2, and 1 μmol/L). Because TSA is unstable in water and degrades easily, fresh TSA was added every 24 h. After 72 h, the cells were washed and fresh medium was added, followed by 20 μl Cell Titer 96®AQ_ueous_ One Solution (Promega, USA) to each well. The cells were then incubated for 1~4 h at 37°C in a humidified atmosphere with 5% CO_2_. The absorbance at 490 nm was measured using a plate reader. The blank control was prepared using untreated cells. Each treatment was performed in triplicate.

### Radioresistance analysis

Sorted SP and non-SP cells as well as unsorted HeLa cells were transferred to 25-cm^2^ flasks and incubated in DMEM with 10% FBS at 37°C with 5% CO_2_ for 24 h. The flasks were placed on a linear accelerator Clinac 600C/D (VARIAN, USA) with a fixed source skin distance at 100 cm and X-ray irradiation at 4 Gy/min. The flasks were covered with a 1.5 cm-thick wax film during X-ray irradiation. Three treatments were carried out at 1, 2, and 4 Gy, respectively. The controls were not exposed to X-rays and were cultured under normal conditions. After X-ray irradiation, cells were digested with trypsin and resuspended in DMEM with 10% FBS for cell counting. The cells were transferred to 96-well plates (2000 cells/well) and cultured for 7 days. The medium was replaced every other day. To generate a radiation survival curve, the SF of cells at each radiation dose was normalized to that of the sham-irradiated control (ibid).

### Statistical analysis

We run the SPSS 19.0 statistical software to process the data and applied the t-test and Fisher's exact test to evaluate if significant differences exist between groups according to the criterion (P < 0.05).

## Abbreviations

CSCs: Cancer stem cells; SP: Side population; ABC: ATP-binding cassette; BCRP: Breast cancer resistance protein; TSA: Trichostatin A; MDR: Multidrug resistance; DMEM: Dulbecco's modified Eagle's medium; FBS: Fetal bovine serum; PBS: Phosphate buffered saline; EDTA: Ethylene diamine tetraacetic acid; FACS: Fluorescence-activated cell sorting; ALDH: Aldehyde dehydrogenase; NOD/SCID: Non-obese diabetic/severe combined immunodeficiency.

## Competing interests

The authors declare that there are no conflicts of interest.

## Authors’ contributions

WLH: Conceived and designed the experiments; QWJ: Performed the experiments and drafted the manuscript; ZC: assisted in designing the experiment and drafted the manuscript; LN: assisted in the laboratory studies; ZLJ: assisted in designing the experiment; LXP and YWD: participated in the coordination of the study. All authors read and approved the final manuscript.

## Authors’ information

Wenjuan Qi and Chao Zhao Co-first author.
